# P-727. Analyzing the Impact of Paid Sex Work on Gender Differences in Sexual Partner Counts: A Multi-Country African Study

**DOI:** 10.1093/ofid/ofaf695.938

**Published:** 2026-01-11

**Authors:** Jacob R Miller, Kathryn Risher

**Affiliations:** Pennsylvania State University, Hershey, PA; Pennsylvania State University, Hershey, PA

## Abstract

**Background:**

Gender differences in reported number of sexual partners are observed consistently across many African nations, with males consistently reporting significantly more lifetime sexual partners than females. While there has been qualitative work to explore factors driving these disparities, national-level quantitative assessments of these factors are limited. The purpose of this study was to investigate the role of engagement with paid sex workers in explaining differences in lifetime sexual partners between men and women.

**Table 1.**
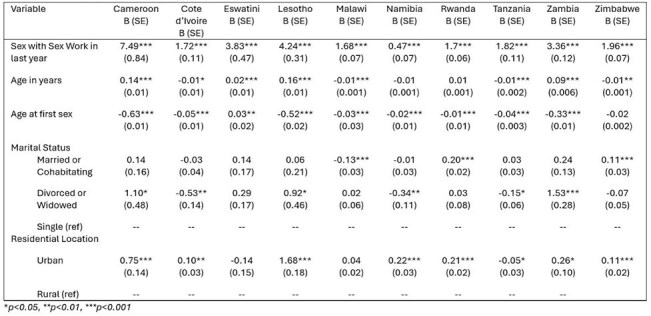
Multivariate linear regression models predicting number of lifetime sexual partners among males compared to females in 2020-2022.

**Table 2.**
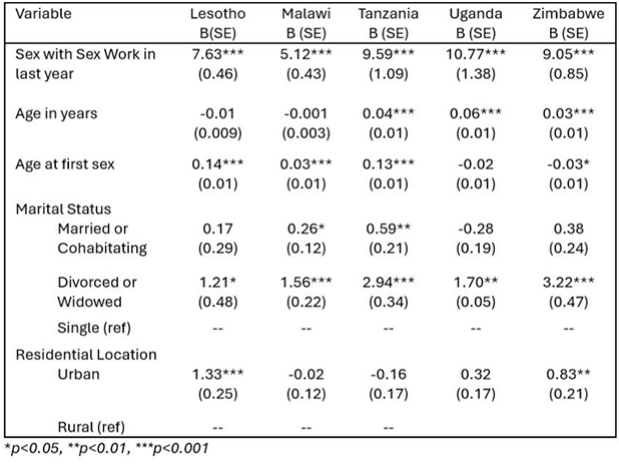
Multivariate linear regression models predicting number of lifetime sexual partners among males compared to females in 2020-2022

**Methods:**

We used data from the Population-based HIV Impact Assessment (PHIA) collected from 10 African nations with historically high HIV incidence. We used linear regression models to examine relationships between reported use of paid sexual services and number of lifetime sexual partners, controlling for age, age at sexual debut, gender, rural/urban residence, and marital status.

**Results:**

Engagement with paid sex workers was associated with having significantly more sexual partners among men compared to females in each nation, however the magnitude of the association varied between nations. Data collected between 2017-2018 in Cameroon, Lesotho, and Zambia, for example, showed that men who participated in paid sex had an average of 7.5, 4.2, and 3.3 (p< 0.001) more lifetime sexual partners than the average female. Data from 2022 demonstrated an increase in the number of sexual partners among men who engaged with sex workers relative to women in some nations, as high as 10.7 (p< 0.001) in Uganda and 9.6 (p< 0.001) in Tanzania.

**Conclusion:**

These findings suggest that the use of paid sexual services is a key contributor to observed gender disparities in sexual partner numbers, and could possibly elevate the risk of contracting HIV or other STI’s in those who participate in paid sex work. This study highlights the need for targeted interventions focusing on sex workers and their partners, especially in locations where optimized allocation of limited resources is important. Such interventions might include enhanced HIV testing services among sex workers and their partners, or improved access to sexual health educational resources. Appropriately tailored efforts may more effectively address drivers of sexual health disparities and guide the use of public health resources.

**Disclosures:**

All Authors: No reported disclosures

